# ASO Author Reflections: Frequent Relapses Prior to the Start of Adjuvant Therapy in Stage IIIB/C Melanoma

**DOI:** 10.1245/s10434-019-07665-5

**Published:** 2019-08-08

**Authors:** Martine Bloemendal, Wouter W. van Willigen, Kalijn F. Bol, Johannes H. W. de Wilt

**Affiliations:** 1 Department of Medical Oncology, Radboud University Medical Center, Nijmegen, The Netherlands; 2 Department of Tumor Immunology, Radboud Institute for Molecular Life Sciences, Nijmegen, The Netherlands; 3 Department of Surgery, Radboud University Medical Center, Nijmegen, The Netherlands

## Past

For decades, surgery has been the mainstay of treatment of melanoma with regional lymph node involvement; however, after resection, the risk of recurrence and metastatic melanoma is significant. Despite major developments in systemic treatment, advanced melanoma still leads to death in the majority of patients. Therefore, adjuvant treatment following surgery to prevent recurrent disease has been widely investigated.^1^ Recently, the approval of adjuvant treatment with immune checkpoint inhibitors and targeted therapy by both the US Food and Drug Administration (FDA) and the European Medicines Agency (EMA) drastically changed the management of lymph node-involved melanoma. Recurrent disease predominantly occurs in the first years after lymph node resection^2^; however, no data on the risk of relapse as early as at the start of adjuvant therapy have been published. Consequently, it was not known whether restaging after surgical resection is appropriate. Therefore, we recorded relapses during screening for eligibility for an adjuvant phase III trial.

## Present

Our data reveal that an important portion of melanoma patients has detectable disease prior to the start of adjuvant therapy, despite a recent surgical resection and preoperative exclusion of distant metastases with adequate imaging techniques.^3^ We analyzed the results of 120 stage IIIB/C melanoma patients (American Joint Committee on Cancer 7th edition) screened for eligibility for experimental adjuvant therapy in a phase III trial. Of those patients, 22 (18%) showed an early recurrence prior to the start of systemic therapy. In all early relapsed patients, metastases had been excluded preoperatively, mainly by using an 18F-fluorodeoxyglucose positron emission tomography/computed tomography scan. The majority of recurrences was discovered within 2 months from surgery. Discovery of an early relapse yields important prognostic information and might change the clinical treatment plan.

## Future

According to our data, rapid recurrences occurred in subgroups with relatively high- and low-risk features. We therefore recommend to restage all IIIB/C melanoma patients after surgery prior to the start of adjuvant therapy; however, stage IIIB/C melanoma is a highly heterogeneous disease. Selecting patients at minimal risk of recurrence prior to adjuvant therapy reduces unnecessary imaging and thereby lowers radiation exposure and improves cost effectiveness. Additionally, comparison of the frequency of early relapses in patients referred for trial participation with patients referred for regular adjuvant treatment is of interest. Furthermore, there is a lack of evidence regarding the role of incorporating imaging techniques in the follow-up of stage III melanoma patients in the current therapeutic era.^4^ Therefore, prospective studies comparing various imaging techniques and intervals might be helpful to the development of consensus regarding the timing and frequency of imaging prior, during, and after adjuvant therapy.
